# Long-Term Outcomes of Laparoscopic Gastric Plication for Treatment of Morbid Obesity: a Single-Center Experience

**DOI:** 10.1007/s11695-022-06217-3

**Published:** 2022-08-12

**Authors:** Mohamed Abdelgawad, Ahmed Elgeidie, Mohamed El Sorogy, Mohamed Elrefai, Hosam Hamed, El-Sayed Abou El-Magd

**Affiliations:** 1 Faculty of Medicine, Mansoura University, Mansoura, Egypt; 2 Department of General Surgery, Faculty of Medicine, Gastrointestinal Surgical Center GISC, Mansoura University, Al Dakahlia Governorate, Gehan Street, Mansoura, 35511 Egypt

**Keywords:** Morbid obesity, Bariatric surgery, Laparoscopic gastric plication, Weight loss

## Abstract

**Background:**

Although laparoscopic gastric plication (LGP) has been mentioned in many studies, its practice has not yet been standardized. In addition, the outcomes remain conflicting, especially long-term ones. This study was conducted to elucidate the long-term consequences of LGP.

**Methods:**

Retrospective analysis of patients with obesity underwent LGP at our institution between March 2010 and September 2014. Data were prospectively collected from our database.

**Results:**

Of the 88 consecutive patients in the study period between 2010 and 2014, follow-up data out to 6 years was available in 60 LGP patients (68.18%). The mean age of the included patients was 41.3 ± 10 years. A total of 81.7% were females. We observed a significant BMI reduction out to 2 years (*p* < 0.001), a plateau at 3 and 4 years, and a significant BMI increase at 6 years (*p* < 0.01). %TWL at 2 years was 21.14% and 12.08% at 6 years. Weight regain was observed in 35 patients at 6 years to reach a rate of 58.3%. Predictors for weight regain at 6 years were disrupted plication fold, increased hunger, and non-adherence to regular exercise. The diabetes improvement rate was 66.6% at 6 years. There were 14 re-operations (23.3%): 1 emergency (1.6%) and 13 (21.6%) elective. There was no mortality.

**Conclusion:**

At the 6-year follow-up visit, LGP has a much less durable effect on weight loss with a % EWL of 32% and a weight regain of 58.3% resulting in a high rate of revisions.

**Graphical abstract:**

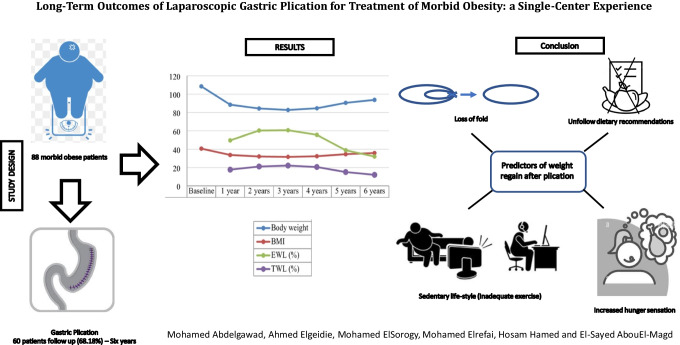

## Introduction

Severe obesity has been a great health burden for developed and developing countries [1, 2], including Egypt. Bariatric surgery has been proven to be the ideal solution for that problem, as it could achieve durable weight loss, resolution, and/or improvement of obesity-associated medical problems and reduction of health care services [3–5].

The laparoscopic greater curve plication (LGP) procedure was originally described in 2007 by Talebpour and Amoli as a cheap alternative for laparoscopic sleeve gastrectomy (LSG) [6]. This procedure could be suitable for patients who do not want to change their body physiology by preserving their stomach[7].

This procedure has gained popularity because it is more conservative, less invasive, has reversible potency, and has minimal leakage risk [8]. Although it is a safe, simple, and cheap procedure, its long-term weight loss outcomes have been questioned [3].

In 2017, our group published 15-month findings of a prospective, consecutive case series of 88 patients with severe obesity who underwent LGP as a step towards standardization of this bariatric procedure [7]. Herein, we describe the long-term outcomes for this LGP cohort through 6 years of follow-up.

## Patients and Methods

### Study Design and Patient Inclusion

This is a retrospective cohort study conducted for patients with severe obesity who underwent laparoscopic gastric plication procedure at Gastrointestinal Surgical Center (GISC) between March 2010 and September 2014.

We included the same patients in our previous short-term study [7], who showed regular follow-up during the scheduled visits and complied with instructions commenced after discharge. On the other hand, patients lost at follow-up or showed hesitancy in following post-operative instructions were excluded.

Finally, 60 patients that fulfilled the inclusion criteria were included in the current study. All cases were subjected to detailed history taking, clinical examination, and routine laboratory investigations in addition to upper GI endoscopy.

All patients had signed informed written consent, and the study gained approval from the local ethical committee.

### Surgical Technique

The laparoscopic procedure was performed as described by Ibrahim et al. [7]. In an anti-Trendelenburg French position, trocar design was as follows: supraumbilical camera port, two working ports to the right and left of the midline, and an assistant port for liver retraction. Devascularization of the greater gastric curve was started 6 cm proximal to the pylorus using harmonic or ligasure hemostatic devices. Care was taken to take the bites 2 cm away from the gastric wall to avoid thermal injury and potential leakage.

Devascularization continued until reaching a point 2 cm from the cardio esophageal junction. Before plication, a 38-Fr bougie was inserted for proper calibration. Plication was performed in two rows (via two anterior and two posterior bites). It was performed using prolene, ethibond sutures, or both.

### Post-operative Care and Follow-up Program

Patients were discharged to the internal ward, where oral fluid was allowed on the 1st postoperative day. Proton pump inhibitors, prokinetics, and antispasmodics were commenced if needed. Patients were allowed to take a fluid diet during the initial 2 weeks, semisolids for the subsequent 4 weeks, followed by a regular diet.

Regular follow-up visits were scheduled at 1, 3, 6, and 12 months, then yearly after the operation. During these visits, all patients were clinically and biochemically assessed. Weight, BMI, the percentage of total weight loss (% TWL), and the percentage of excess weight loss (%EWL) were calculated and recorded during these visits.

Postoperative de novo gastroesophageal reflux disease (GERD) was defined as the postoperative development of reflux symptoms in patients not complaining of it before [9], and it was confirmed by an upper GI endoscopy.

At the 6-year follow-up visit, an endoscopic evaluation of patients was done. Plication fold was graded according to the presence of fold continuity: grade A for prominent plication fold and grade B for partial or completely disrupted plication fold.

Postoperatively, patients were asked to rate their hunger sensation between meals using a ten-grade scale, where 0 was no hunger, and 10 was extreme hunger sensation [19].

Patient compliance to regular physical exercise (based on patients’ subjective self-reported exercise diary) and diet regimen applied by the nutritionist was recorded.

Weight regain was defined as > 25% of EWL from nadir weight [10]. Inadequate weight loss was defined as %EWL less than 50% in the first 18 months postoperatively [26, 27].

We analyzed the risk factors of weight regain at the 6-year follow-up.

### Outcomes and Data Collection

The primary study outcome was to assess the evolution of BMI and %TWL over 6 years in 60 LGP patients.

The percentage of total weight loss was calculated as (%TWL: calculated as [baseline absolute weight − follow-up absolute weight]/[baseline absolute weight] × 100) [3].

Assessments also included the percentage of excess weight loss (%EWL: calculated as [preoperative weight − current weight]/[preoperative weight − ideal weight] × 100 relative to the 1983 Metropolitan Life Insurance tables).

Specific focus was given to changes from baseline in weight, %TWL, excess weight, and BMI at 2, 5, and 6 years. Weight-loss outcomes were assessed at 2, 5, and 6 years. Also, predictors of weight regain at the 6-year follow-up were assessed. Secondary outcomes were co-morbidity improvement, complications including de novo GERD, and reoperation rate.

### Statistical Analysis

The collected data were coded, processed, and analyzed using the SPSS (Statistical Package for Social Sciences) version 27 for Windows® (IBM SPSS Inc, Chicago, IL, USA). Data were tested for normal distribution using the Shapiro Walk test. Parametric quantitative data were expressed as mean ± SD (standard deviation) and/or median and range. Qualitative data were represented as frequencies and relative percentages.

Repeated measures ANOVA was used to compare parametric data at more than two-time points, while paired samples *t*-test was used to compare parametric data at two-time points. Significance test results are quoted as two-tailed probabilities. For all the tests mentioned above, the significance level was tested, expressed as the probability of *p*-value with considering *p*-value significant if < 0.05. Univariate and multivariate regression analyses were used to assess the dependent and independent predictors of weight regain at the 6-year follow-up.

## Results

### Baseline Patient Characteristics

Of the 88 consecutive patients, follow-up data out to 6 years was available in 60 LGP patients (68.18%), and follow-up rate at 1, 2, 3, 4, 5, and 6 years was recorded. The mean age of the included patients was 41.3 ± 10 years. A total of 81.7% were females. They had a mean BMI of 40.7 ± 7.7 kg/m^2^.

T2DM was the most prominent-associated medical problem 21(35%), followed by hypertension 9 (15%), and sleep apnea 6 (10%). Additionally, four patients (6.7%) had gallstones diagnosed preoperatively, and all patients were subjected to concomitant laparoscopic cholecystectomy with LGP.

Regarding previous weight loss attempts, dieting was tried by 50% of patients, while only one patient had a previous gastric balloon. The previous data are summarized in table (1).

### Operative Data and Hospital Stay

All operations were performed laparoscopically. The mean operative time was 162.58 ± 10 min. There were no conversions during any of the surgeries done. Concomitant cholecystectomy was done in 4 patients (6.7%). Oral intake was allowed on the first postoperative day in all patients. Mean hospitalization was 2.3 ± 0.5 days (range 1–3).

### Weight Outcomes

**Fig. 1:**
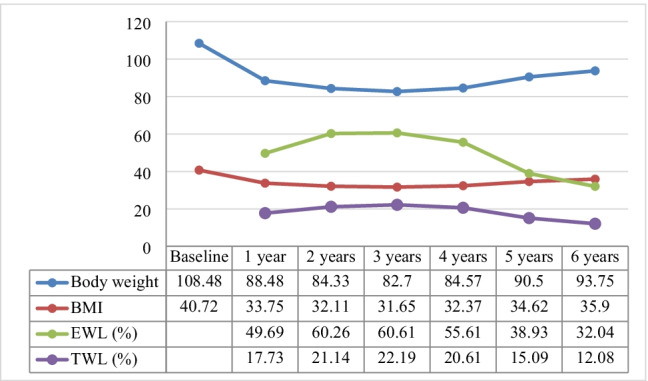
Body weight, body mass index (BMI, kg/m^2^), % TWL, and excess weight loss (%EWL) through 6 years after LGP

**Fig. 2:**
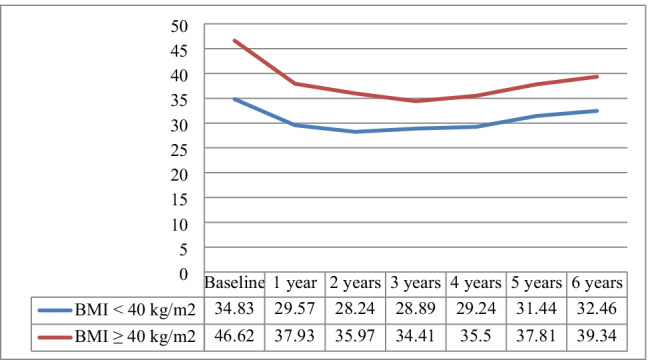
Body mass index (BMI, kg/m^2^) evolution of patients with baseline < 40.0 vs. ≥ 40.0 BMI

As shown in Fig. 1, the mean BMI was reduced from 40.72 ± 7 to 33.75 ± 5 kg/m^2^ at 1 year (*p* < 0.001). The significant downward BMI trend persisted for 2 years, 32.11 ± 5 kg/m^2^ (*p* < 0.001). A relative plateau occurred from 2 to 4 years following LGP, with no significant change in mean BMI. However, from 4 to 6 years, a moderate but significant increase in BMI was observed, 35.90 ± 6 kg/m^2^ (*p* < 0.001). This trend was more evident in patients with a baseline BMI > 40 (Fig. 2).

**Fig. 3:**
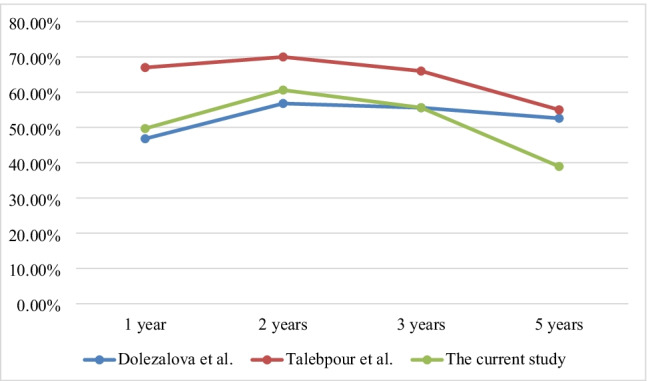
Comparison of % of EWL of our study with LGP results from Talebpour et al. [8] and Doležalova et al. [3]

For comparative purposes, current LGP %EWL data are integrated into Fig. 3 with Talebpour et al.’s [8] and Doležalova et al.’s [3] 5-year LGP follow-up data.

**Fig. 4:**
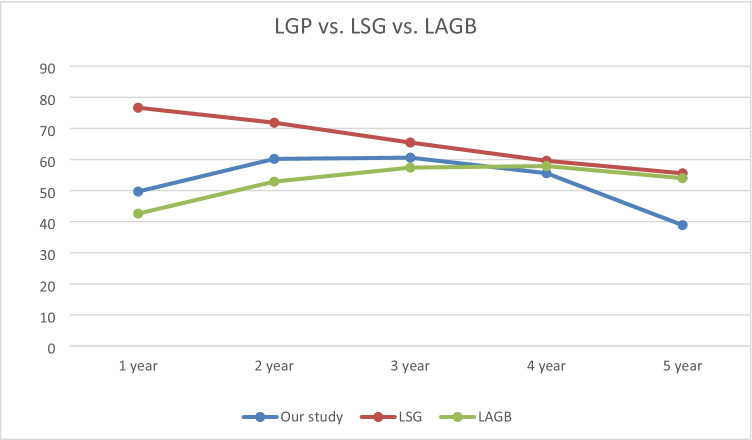
Comparison of % of EWL of our study with other restrictive procedures as LSG and LAGB

**Fig. 5:**
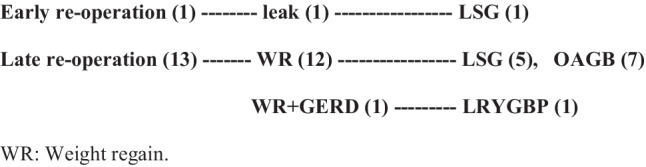
Rate and reasons for re-operation after LGP. WR weight regain

Also, our LGP data regarding %EWL are integrated into Fig. 4 and 5 with other restrictive procedures such as laparoscopic adjustable gastric banding (LAGB) (as projected by O’Brien et al.’s meta-analysis [11] and with laparoscopic sleeve gastrectomy (LSG) as projected by the combined results of Golomb et al., Lemanu et al., and Sepúlveda et al. [12–14].

At 6 years, obesity indicators remained significantly reduced relative to baseline measures. For example, absolute weight was 93.75 ± 16 compared to 108.48 ± 23 kg at baseline, a mean reduction of 14.73 ± 17 kg (*p* < 0.001). However, a significant amount of weight regain was evident: the mean %TWL value after 6 years was 12.08 ± 13.1% compared to 21.1 ± 8.9% after 2 years. Similarly, the %EWL at 6 years was 32.04% compared to the 2-year level of 60.2%, as shown in table (2).

### Weight Regain

During the scheduled follow-up period, inadequate weight loss was observed in 11 (18.3%) patients. Weight regain was observed in 35 patients at 6 years to reach a rate of 58.3%. Some variables were evaluated to define risk factors for weight regain after LGP at 6 years (Table 1, 2, 3, 4): age, sex, weight, BMI, associated medical problems, suture used for the plication, distance from the pylorus, post-operative hunger sensation, de novo GERD, and patient compliance or adherence to diet and regular exercise.

**Table 1:** Baseline patient characteristics

	Total number of cases = 60	
	Number	Percent
Age/years (mean ± SD (min–max))	41.3 ± 10 (16–58)	
Gender Male Female	11 49	18.3 81.7
Co-morbidities		
DM	21	35
HTN	9	15
OSAS	6	10
Hyperlipidemia	5	8.3
Osteoarthritis	5	8.3
Gallstones	4	6.7
Weight-loss attempts Diet Intragastric balloon	30 1	50 1.7
Weight/kg (mean ± SD (min–max))	108.48 ± 23.8	
Height/cm (mean ± SD (min–max))	162.02 ± 8.8	
BMI/kg/m^2^ (mean ± SD (min–max))	40.72 ± 7.76 (25.7–64.8)

**Table 2:** Weight loss at 2, 5, and 6 years in the studied patients

	Baseline	2 years	Change at 2 years	5 years	Change at 5 years	6 years	Change at 6 years
Weight	108.48 ± 23	82.70 ± 12	25.78 ± 17	90.50 ± 14	17.98 ± 17	93.75 ± 16	14.73 ± 17
			*p* < 0.001*		*p* < 0.001*		*p* < 0.001*
%TWL		21.14		15.09		12.08	
BMI	40.72 ± 7	31.65 ± 5	9.07 ± 6	34.62 ± 5	6.10 ± 6	35.90 ± 6	4.83 ± 6
			*p* < 0.001*		*p* < 0.001*		*p* < 0.001*
%EWL		60.2		38.93		32.04

**Table 3:** Univariate and multivariate regression analysis of predictors of weight regain at 6 years (*n* = 35)

Variables	Univariate analysis	Multivariate analysis		
		OR	95% CI for OR	*p*-value
Age	0.697			
Male gender	0.372			
Pre-operative weight				
Weight	0.366			
BMI > 40	0.847			
Co-morbidities				
DM	0.511			
HTN	0.584			
OSAS	0.664			
De novo reflux	0.812			
Disrupted plication fold	0.010*	1.424	1.108–1.718	0.032*
Suture used in the plication				
Ethibond and prolene	0.262			
Prolene only	0.458			
Distance from pylorus	0.711			
Hunger score	< 0.001*	1.952	1.646–2.354	0.001*
Patient, non-adherence (diet and regular exercise)	0.002*	1.380	1.108–1.946	0.010*
%EWL at 1 year	0.054			

*OR* odds ratio, *CI* confidence interval

^*^Statistically significant (*p* < 0.05)

**Table 4:** Post-operative de novo reflux in the studied patients

	All patients (*n* = 60)	Odds ratio	*p*-value
Pre-operative	0 (0%)	-	-
3 months	11 (18.3%)	2.22	0.001
6 months	9 (15%)	1.64	0.003
1 year	6 (10%)	1.29	0.014
2 years	4 (6.6%)	1.15	0.046
3 years	2 (3.3%)	1.06	0.157
4 years	2 (3.3%)	1.06	0.157
5 years	2 (3.3%)	1.06	0.157
6 years	2 (3.3%)	1.06	0.157

At 6 years, endoscopic evaluation was done for 50 (83.3%) patients for the condition of the plication fold, and we found a partially or completely disrupted fold in 40 (80%) patients. The risk factors for weight regain at 6 years after LGP, disrupted plication fold, higher hunger scores, and non-adherence to regular exercise and diet were significant factors in the univariate analysis.

On the multivariate one, disrupted plication fold, higher post-operative hunger sensation, and non-adherence to regular exercise and diet were independent factors associated with weight regain after LGP, as shown in table (3).

### Associated Medical Problem Improvement

At 2 years following LGP, 18 of 21 preoperatively diabetic patients (85.7%) experienced surgically induced improvement. Three patients (14.3%) showed no significant change from baseline. At 6 years, the improvement rate declined to 66.6% (14/21). Regarding patients with HTN, 77.7% described improvement after LGP at 2 years, while this improvement declined to 66.6% at 6 years.

### Complications and Re-operations

There was no mortality in our series. Early minor post-operative complications such as nausea, vomiting, and epigastric pain occurred in 5 of 60 cases (8.3%). Those patients had been successfully treated with intravenous fluids, parenteral proton pump inhibitors, and antiemetics during their hospital admission, and they were discharged once they tolerated oral drinks.

De novo GERD showed a significant increase in incidence compared to the baseline value, as no patients complained of GERD symptoms preoperatively. GERD symptoms were experienced by 18.3%, 15%, and 10% of cases at 3-, 6-, and 12-month follow-up visits, respectively. This incidence decreased to 3.3% at the last follow-up period, as shown in table (4).

Interestingly, after the endoscopic evaluation of 50 patients, at 6 years, only 4 patients had erosive esophagitis, 3 of them had grade A reflux esophagitis, and the other one had grade B reflux esophagitis.

All of them were successfully treated with proton pump inhibitor (PPI) drugs, except one patient managed by laparoscopic Roux en Y gastric bypass (LRYGBP) due to refractory GERD with weight regain.

Only one patient (1.6%) presented with a major complication and required emergency reoperation due to early leakage from the proximal one-third of the plicated gastric fundus, mostly due to thermal injury during devascularization. On emergency laparoscopic reoperation, about 0.5 cm defect at the upper third was managed by the undoing of the plication and conversion into LSG.

Also, in the later stages of 6-year follow-up, 21.6% (13/60) elective reoperations were performed due to weight regain and GERD. This included LSG in 5 patients, laparoscopic one anastomosis gastric bypass (OAGB) in seven patients, and LRYGBP in one patient. The overall reoperation rate was 23.3% (emergency reoperation in one patient while elective reoperation in13 patients), as shown in Fig. (5).

## Discussion

Despite many studies confirming the benefits of laparoscopic gastric plication in weight loss, the American Society for Metabolic and Bariatric Surgery (ASMBS) considered it an investigational procedure that should be performed under a study protocol [15].

There is scarce long-term evidence about the follow-up of 5 or more years for plication. Only Talebpour [8] and Dolezalova-Kormanova had long-term results [3].

Our study was conducted to elucidate the long-term outcomes of the laparoscopic gastric plication procedure. Our findings revealed a significant increase in %EWL with mean values of 49.6 and 60.2 at 1- and 2-year follow-up visits, respectively.

However, subsequent long-term follow-up revealed a significant decrease in% EWL, with a mean value of 55.6, 38.9, and 32.04 at 4, 5, and 6 years, respectively.

Talebpour et al. reported that %EWL had mean values of 67% and 70% at 1 and 2 years, respectively. However, subsequent follow-up revealed a decrease in EWL, with mean values of 66%, 55%, and 42% after 3, 5, and 10 years, respectively [8].

Also, Doležalova-Kormanova reported that the mean values of %EWL were 46.8% and 56.8% at 1 and 2 years, respectively. However, subsequent follow-up revealed that the mean %EWL was 55.6%, 54.1%, and 52.6% at 3, 4, and 5 years, respectively [3].

Our 5-year %EWL results were lower than those of Talebpour et al. (55.0% EWL) and Doležalova et al. (52.6% EWL), the only two groups that have published 5-year or greater LGP outcomes [3].

Furthermore, our series observed a high weight regain rate of 58.3% at 6 years of follow-up. On the other hand, Talebpour et al. revealed a weight regain rate of 5.5%, 31%, and 42% after 4, 8, and 10 years of follow-up, respectively [8], while Donazalova et al. reported a lower rate of 9.2% at the 5-year follow-up [3].

Atrophy of the plicated gastric portion and the gradual extension of the elastic gastric wall are two major causes of the limited long-term effect of the gastric plication procedure [19].

Unfolding of the plicated greater curvature of the stomach was observed either on long-term endoscopic evaluation [16, 20, 21] or during revision surgeries [3, 21].

In our study, at the 6-year follow-up, an endoscopic evaluation of 50 patients was done to assess the plication fold condition. We found disruption of the plication fold either partially or completely in 40 (80%) patients.

Also, we found that disruption of the plication fold was one of the main predictors of weight regain.

Hence, the preservation of long-term weight reduction with LGP appears to be a challenge for such patients.

Bradnova et al. investigated the effect of laparoscopic gastric plication on type 2 diabetes. During the early 6 months after the operation, the authors concluded that plication induces significant weight loss and improves the metabolic profile of such patients [22].

The long-term effects of LGP on T2DM have not been well studied. Doležalova et al. provide the only other group that has published 5-year or greater LGCP T2DM outcomes. They reported that LGP-induced T2DM improvement was observed in 89.7 and 65.5% of patients at 2- and 5-year follow-ups, respectively [3].

In our series, LGP-induced T2DM improvement was 85.7% and 66.6% at 2- and 6-year follow-up visits, respectively. This high improvement rate was noted as most diabetic patients have sustained weight loss. This explains a good relation between sustained weight loss and DM improvement.

Our findings showed a significant increase in the incidence of postoperative de novo GERD compared to the baseline value. GERD symptoms were experienced by 18.3%, 15%, 10%, and 6.6% of cases at 3-, 6-, 12-, and 18-month follow-up visits, respectively (*p* < 0.05). This incidence decreased to 3.3% at the last follow-up period.

In the same context, other authors reported that GERD was one of the causes of revision after gastric plication. The authors reported that two cases experienced GERD symptoms and gastric prolapse after 6 months. One patient was managed by sleeve gastrectomy, whereas the other was managed by fundus resection [23].

One could explain the decrease in the incidence of such complications at subsequent follow-up visits by two facts; optimum weight loss achieved at this follow-up could help decrease GERD symptoms. The second is the supposed relative dilatation of the plicated stomach which might decrease the intragastric pressure leading to decrease GERD manifestations.

In the systematic review conducted by Abdelbaki et al., 8% of the patients suffered problems, with individual author complication rates ranging from 7 to 15.3%. All studies reported mild to moderate nausea and vomiting, which usually subsided within 1–2 weeks. Twenty patients (6.5%) were readmitted, with 14 (4.6%) requiring reoperation, largely due to stomach blockage [24].

Although this procedure seems safe compared to the laparoscopic sleeve gastrectomy, as reported by previous studies [17, 25], the risk of bleeding or perforation could not be eliminated.

In our study, we encountered a case of gastric leakage, successfully managed by the laparoscopic unfolding of the plication and conversion into sleeve gastrectomy. 

In fact, the overall rate of revision after laparoscopic gastric plication is high. Albanese et al. reported that 55.57% of gastric plication cases underwent surgical revision after a mean time of 18 ± 8 months [23]. Others reported an elective reoperation rate of 3.3% (8/244) after 5 years of follow-up [3].

In our series, at the 6-year follow-up, the rate of elective reoperation was 21.6% (13/60), and the commonest cause of revision was weight regain.

Interestingly, we evaluated different factors to define the main predictors for weight regain after LGP at 6 years. We found that disrupted plication fold, higher postoperative hunger sensation, and non-adherence to diet and regular exercise were independent factors associated with weight regain after LGP. Gudaityte et al. agreed with our finding as higher post-operative hunger sensation was found to be an independent factor associated with unsatisfactory weight loss after LGP [19].

All in all, one could see that LGP is associated with poor weight loss outcomes in the long term. Although desirable outcomes could be achieved in patients with good post-operative compliance regarding diet and exercise, these factors could not be predicted before the operation.

## Limitations of the Study

Our study has several limitations; it is a single-center study with relatively small sample size. However, it has an impressive 6-year follow-up. It would be a valuable addition to the scanty literature on the long-term impact of laparoscopic gastric plication on patients with severe obesity**.**

We did not use any GERD-specific questionnaires to define GERD. Also, no valid questionnaires were used to measure patient compliance.

Further studies with a larger sample size and longer follow-ups are needed to discover the durability of the LGP procedure.

## Conclusion

After 6 years, LGP has a much less durable effect on weight loss with a mean %EWL of 32% and weight regain of 58.3%, resulting in a high rate of revisions (13/60), reaching about 21.6%.
